# Implementation of a novel malaria management strategy based on self-testing and self-treatment in remote areas in the Amazon (Malakit): confronting a-priori assumptions with reality

**DOI:** 10.1186/s12889-022-12801-0

**Published:** 2022-04-15

**Authors:** Muriel Suzanne Galindo, Yann Lambert, Louise Mutricy, Laure Garancher, Jane Bordalo Miller, José Hermenegildo Gomes, Alice Sanna, Cassio Peterka, Hedley Cairo, Helene Hiwat, Antoine Adenis, Mathieu Nacher, Martha Cecilia Suárez-Mutis, Stephen Vreden, Maylis Douine

**Affiliations:** 1 Centre d’Investigation Clinique Antilles-Guyane, Inserm CIC 1424, Cayenne Hospital, Cayenne, French Guiana; 2 The Ink Link, Paris, France; 3 DPAC Fronteira, Oiapoque, Brazil; 4 National Malaria Control Program, Ministry of Health of Brazil, Brasília, Brazil; 5 Malaria Program, Ministry of Health of Suriname, Paramaribo, Suriname; 6 TBIP, Université de Guyane, Université de Lille, CNRS, Inserm, Institut Pasteur de Lille, U1019-UMR9017-CIIL Centre d’Infection Et d’Im munité de Lille, Cayenne, French Guiana; 7 Laboratory of Parasitic Diseases, Institute Oswaldo Cruz/Fiocruz, Rio de Janeiro, Brazil; 8 Foundation for the Advancement of Scientific Research, Paramaribo, Suriname

**Keywords:** Border malaria, Mining population, Remote health, Process evaluation, Implementation outcomes

## Abstract

**Background:**

A novel strategy to combat malaria was tested using a methodology adapted to a complex setting in the Amazon region and a hard-to-reach, mobile community. The intervention strategy tested was the distribution, after training, of malaria self-management kits to gold miners who cross the Surinamese and Brazilian borders with French Guiana to work illegally in the remote mining sites in the forest of this French overseas entity.

**Main text:**

This article aims at presenting all process and implementation outcomes following the Conceptual Framework of Implementation Fidelity i.e. adherence, including content and exposure, and moderators, comprising participant responsiveness, quality of delivery, facilitation strategies, and context. The information sources are the post-intervention survey, data collected longitudinally during the intervention, a qualitative study, data collected during an outreach mission to a remote gold mining site, supervisory visit reports, in-depth feedback from the project implementers, and videos self-recorded by facilitators based on opened ended questions.

As expected, being part of or close to the study community was an essential condition to enable deliverers, referred to as “facilitators”, to overcome the usual wariness of this gold mining population. Overall, the content of the intervention was in line with what was planned. With an estimated one third of the population reached, exposure was satisfactory considering the challenging context, but improvable by increasing ad hoc off-site distribution according to needs. Participant responsiveness was the main strength of the intervention, but could be enhanced by reducing the duration of the process to get a kit, which could be disincentive in some places. Regarding the quality of delivery, the main issue was the excess of information provided to participants rather than a lack of information, but this was corrected over time. The expected decrease in malaria incidence became a source of reduced interest in the kit. Expanding the scope of facilitators’ responsibilities could be a suitable response. Better articulation with existing malaria management services is recommended to ensure sustainability.

**Conclusions:**

These findings supplement the evaluation outcomes for assessing the relevance of the strategy and provide useful information to perpetuate and transfer it in comparable contexts.

**Trial registration:**

ClinicalTrials.gov. NCT03695770. 10/02/2018 “Retrospectively registered”.

**Supplementary Information:**

The online version contains supplementary material available at 10.1186/s12889-022-12801-0.

## Background

Ineffective programs can be well implemented while useful programs can be poorly implemented [1, 2]. Knowing the degree to which an intervention that has been implemented corresponds to the intervention initially designed can be very helpful when assessing the sustainability, applicability, or transferability of a strategy [3, 4].

In any kind of research, experimental design is considered to be the most rigorous methodology to ensure the highest level of evidence [5–7]. In some contexts, however, random allocation of individuals or clusters is not feasible: this may actually be an opportunity in disguise. Indeed, the quest for gold standard methods can overshadow the relevance of a more pragmatic design as well as some of its advantages, such as transferability [3, 8, 9]. Such a context can be found in the Guiana Shield, a part of the Amazon region, and more specifically in French Guiana, a French overseas entity, bordered by Suriname and the Brazilian State of Amapá. The area’s mining potential, inherited from its rich geological history, attracts a highly-mobile and widely-dispersed population, most of whom come from the poorest regions of Brazil. The high risk of exposure to vectors linked to the living and working conditions of these gold miners – long working hours, stagnant water due to alluvial gold mining practices, etc. – is conducive to the spread of malaria, which is endemic in the region, as detailed in the Additional file 1 [10–13].

Major difficulties in reaching isolated areas and the sensitive transborder context involving an illegal migrant population raised serious methodological challenges [14].

Border malaria has long been a problem, notably in South East Asia (on the borders between Myanmar and Thailand and Cambodia and Thailand, for example), where antimalarial resistance has repeatedly emerged in a particular mix of local circumstances [15–17]. Throughout the history of malaria programs, great efforts have been made to target this complex transnational context [17–20]. Furthermore, certain activities, often illegal (guerrilla warfare, logging, mining), have been important drivers of malaria epidemiology. In South America (in Venezuela and Colombia for instance), malaria has been linked to mining or more largely to extractive activities [10, 21–24]. The malaria problem on the Guiana Shield is thus specific, but shares certain characteristics with situations found elsewhere in the world.

An innovative research project called Malakit focused on this neglected population which has been identified as a key host and a barrier to the elimination of the disease [25–27]. This international collaborative project aimed at evaluating the effectiveness of the preventive distribution of self-diagnosis and self-treatment kits, combined with information and training by facilitators, to gold miners*,* at resting sites on the borders, to be used when they were unable to rapidly consult a health care provider [26, 28].

The main objective of the project was to increase the proportion of gold miners who correctly take reliable malaria medication, promptly after the onset of the disease, following a positive diagnosis [25, 26, 29–31].

The communication of results does not always take into account how interventions were implemented and how context affects implementation and outcomes while it is of major importance for measuring the value of public health strategies [4, 32].

The objective of this article is to detail the solutions that were implemented locally and how the planned intervention unfolded in the midst of a challenging context in order to complement effectiveness outcomes and extract applicability and transferability to other contexts with their own set of interventional constraints [3, 33].

## Main text

### Evaluation of the effectiveness of the Malakit intervention

**Fig. 1:**
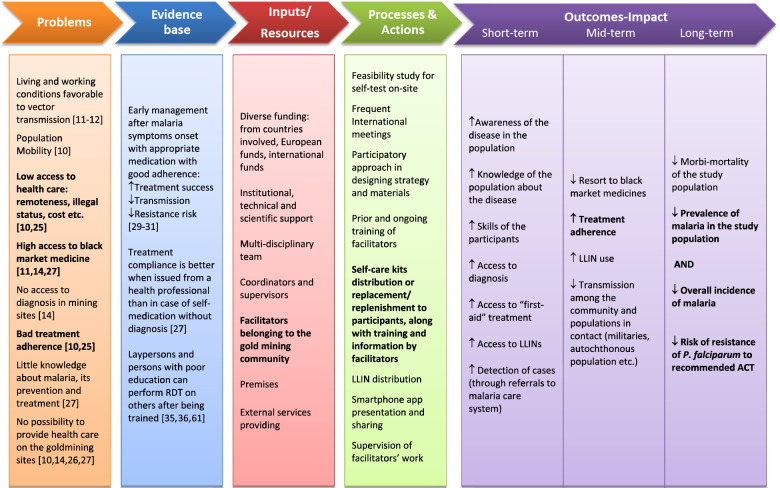
Logic model of the Malakit intervention before the start of the Malakit study [10–12, 14, 25–27, 29–31, 35, 36]. Source: created by the authors

**Fig. 2:**
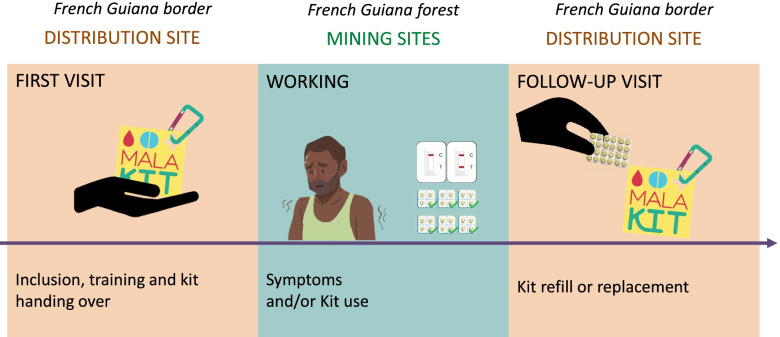
Principle of the Malakit intervention in Suriname and Brazil (April 2018-March 2020). Source: created by the authors

Malakit is a research project involving three countries. The sponsor of the research project was the Hospital of Cayenne (French Guiana) which also had a role in the implementation of the intervention. In Suriname, the National Malaria Program and the Foundation for the Advancement of Scientific Research in Suriname (SWOS) were responsible for the investigation and implementation. In Brazil, the institution involved in investigation was Foundation Oswaldo Cruz (Fiocruz) and the non-government organization (NGO), DPAC fronteira was in charge of the implementation [28]. The context, the content of the intervention, the players, and the steps of the project development phase have been described in previous articles [26, 28, 34]. Figures 1 and 2 describe the logic model of the Malakit intervention and its principle. The study population included individuals over the age of 18 and individuals aged 15–17 with parental consent, who go to French Guiana’s illegal gold mining sites to work, or accompany someone who works there: miner, machine owner, cook, housekeeper, canoe operator, driver, hawkers or shopkeepers/vendors, sex workers, etc., whether their activity is itinerant or fixed.

Properly defining the type of research study carried out is useful, since clarity can help avoid duplications, funding inefficiencies, and difficulties in seeking and understanding information encountered by the end-users of research evidence [37]. The Malakit project, by developing an unprecedented approach to malaria management, can be classified as intervention research. A before-after study design was developed using measurable, realistic, and comparable variables. The main evaluation criterion was based on proportion of gold miners who declared good diagnostic and treatment practices, measured cross-sectional surveys before and after the intervention [26, 28]. To complete these indicators, continuous and longitudinal data collection was implemented to assess the correct use of the kit [34]. Between April 2018 and March 2020, 4,766 kits were distributed to 3,733 participants. Six hundred and thirty one of them returned to a distribution site to answer questions about their experience during follow-up visits, among whom 223 used at least one malakit [38]. The main outcomes were analyzed and published independently of the Malakit implementation evaluation [4, 38].

### Evaluation of Malakit implementation

The boundaries between intervention and implementation research are not always clear and may closely overlap [37]. Indeed, Malakit could also be considered as an implementation of a test-and treat strategy relying on rapid diagnostic tests (RDT) and artemisinin-based combination therapy (ACTs). In support of this, several implementation research outcomes such as coverage or acceptability were also included in the evaluation from the outset. Therefore, the term of type 2 effectiveness-implementation hybrid trial can be applied to the Malakit study [39–41]. No process evaluation plan was elaborated before the launch of the intervention but the need to report on what was delivered and how, as well as on barriers and levers, became evident during the roll-out of the intervention, in order to complement the effectiveness evaluation outcomes and thus improve validity and inform on applicability and transferability.

### Data collection and analysis

Quantitative and qualitative data were collected from the studies performed as part of the Malakit project, i.e. pre- and post-intervention surveys, Malakit intervention study, an independent qualitative study, and as part of a medical outreach mission carried out alongside the French army at a remote gold mining site known as Repentir (see Table 1). The qualitative study aimed at exploring: 1) the opinion, perception and responsiveness of participants, facilitators, as well as key actors who are community members not eligible for the intervention, 2) levers and barriers to the use of the “malakit”, 3) the role of the facilitators, 4) contextual elements [42]. Other sources of information were also used to complete the overall picture (see Table 1). Supervisory visits were carried out in the field by project implementers, among whom members of the sponsor team, to observe first and follow-up visits and to hold discussions with facilitators [28]. Seventeen supervisory visits were carried out in Suriname and 15 in Brazil. The total duration of the interventional research was 24 months in Suriname and 18 months in Brazil, between April 2018 and March 2020. In both countries, the first supervisory visit took place within one month after the project launch and the final visit took place one month before the end of the study, i.e. just when it became impossible to travel due to border closings in the context of the Covid-19 pandemic. In Brazil, supervisory observations were conducted on 21 first visits and 10 follow-up visits. In Suriname, 15 first visits and four follow-up visits were reported, although more were actually observed.

**Table 1:** Methodologies of the studies carried out as part of the Malakit project and of other sources of data and information

Studies				
	**Pre-intervention survey**	**Post-intervention survey**	**Malakit intervention**	**Qualitative study**
**Aim**	To evaluate the effectiveness of the Malakit intervention strategy		To assess the use of malakits by the participants	To evaluate perception and opinion of the intervention, levers, barriers, and opportunities
**Methodology**	Cross-sectional study		Longitudinal data collection	1) on-site observation; 2) semi-structured individual interviews; 3) semi-structured group
**Place of data collection**	Gold miners’ resting sites on the Surinamese and Brazilian borders with French Guiana		Kit distribution sites	One distribution site in Suriname and one distribution site in Brazil
**Timing**	Surinamese border: January-June 2015; Brazilian border: May-June and October-November 2018	October-December 2019	Throughout implementation phase (April 2018—March 2020)	April 2019 and August 2019
**Population**	All the individuals working at illegal gold mining sites in French Guiana		Malakit participants	22 Malakit participants who used the kit, four facilitators in Brazil, two facilitators in Suriname, and six actors from the local community
**Sampling**	Snow-ball effect		Systematic	Convenience sample
**Data collectors**	One physician, one nurse, and one interpreter/interviewer		Nine Malakit facilitators	One external assessor: Professor of social work
**Analysis type**	See references	See references	See references	Thematic analysis method of Mucchielli & Paillé^a^ using a semi-open descriptive coding grid based on ten general codes^b^
**References**	Douine et al., 2017^c^, [25, 27]	[26, 38]	[26, 28, 38]	[42]
**Other information sources**				
	**Medical outreach mission in Repentir**	**Supervisory visits**	**Feedback from the project implementers**	**Videos of facilitators**
**Aim**	To collect data on the knowledge, perception, and reach of the intervention	To assess the delivery of the intervention	To capitalize on the experience of the implementing actors	To present the facilitators’ points of view at the final meeting of the project
**Methodology**	Cross-sectional data collection	Observation of the intervention and interviews with facilitators	In-depth debriefings	Structured questionnaire sent to the facilitators
**Place of data collection**	At a gold mining site in French Guiana located particularly far from the distribution sites	Kit distribution sites	NA	NA
**Population**	People who voluntarily came for health care to the medical consultation point set up on site	Facilitators and study participants	Project implementers (project implementation team and coordination teams in Brazil and Suriname)	Facilitators
**Timing**	June 2019	Throughout implementation phase (April 2018—March 2020)	Throughout implementation phase (April 2018—March 2020)	September—October 2020
**Sampling**	Systematic	NA	NA	NA
**Data collectors**	Two members of the project implementation team	Members of the project implementation team and supervisors from Brazil and Suriname	Project implementation team	Videos (or only audio files) self-recorded by two facilitators in Brazil and five facilitators in Suriname
**Analysis type**	Descriptive analysis using Stata 13	Extraction of information from the supervision reports	Synthesis of information collected from debriefings throughout implementation	Extraction of information from the videos

^a^Paillé P, Mucchielli A. L’analyse qualitative en sciences humaines et sociales. Armand Colin Éditeur. 2016

^b^Miles MB, Huberman AM, Saldana J. Qualitative Data Analysis: A Methods Sourcebook. Third Edition. SAGE Publications Ltd (CA). SAGE Publications; 2014

^c^Douine M. Epidémiologie du paludisme chez les personnes travaillant sur les sites d’orpaillage illégal en Guyane: quels enjeux pour la santé publique? [Internet]. [Cayenne]: Université de la Guyane; 2017. Available from: http://www.theses.fr/s135439

Finally, information were extracted from more informal sources i.e*.* implementer debriefings conducted throughout the project and video self-recorded by facilitators based on a list of questions (Additional file 2).

### Conceptual framework used

A modified version of the Conceptual Framework for Implementation Fidelity was chosen retrospectively to present all these outcomes [43]. Therefore, implementation outcomes (“Adherence”) were separated from process outcomes (“Moderators”). Adherence was subcategorized into “Content” and “Dose/Exposure”. However, strictly speaking, adherence with regard to frequency was not assessed, as there was no determined target value regarding the number of training sessions, visits, or kits distributed, due to the lack of knowledge on population size and flows at this time. The moderators presented are “Participant Responsiveness”, which concerned both participants and intervention deliverers as described by Hasson, 2010, “Quality of Delivery”, and “Facilitation Strategies”, but “Intervention Complexity” was not assessed [44]. One element was added, based on a modified model used by Hasson in 2010 to systematically evaluate the implementation fidelity of complex interventions in health and social care, i.e. “Context” [44]. Factors related to the research setting were integrated in this last aspect. The research questions and the sources of the answers are summarized in Table 2.

**Table 2:** Implementation and process evaluation questions and data sources for answering the questions, based on a modified version of the Conceptual Framework for Implementation Fidelity

Areas to measure	General questions	Specific questions	Question answering data sources
**Evaluation of adherence**			
Content	To what extent was each of the components of the intervention design implemented as planned?	To what extent was the recruitment of deliverers (“Malakit facilitators”) compliant with what was planned?	Feedback from the project implementers
		To what extent was the process of inclusion, training, kit delivery, and follow-up visits implemented as planned?	Supervisory visits, Malakit intervention data
		To what extent were the messages conveyed during training delivered as planned, including use of tools and materials?	Supervisory visits
Dose/exposure (availability of the intervention and reach/coverage)	What proportion of the target population was covered by the intervention?	Was the intervention optimized in terms of availability for potential participants?	Feedback from the project implementers, Malakit intervention data, supervisory visits
		What was the estimated proportion of the target population (recruited at resting sites) who knew about the project and who participated in the intervention?	Post-intervention survey
		What was the penetration of the intervention in very remote areas?	Medical outreach mission in Repentir
**Moderating factors**			
Participant responsiveness (individuals who received the intervention and individuals responsible for delivering it)	What were the engagement and opinions of the participants and deliverers towards the intervention?	How did the participants perceive the fit of the intervention and what was the level of enthusiasm and participation among the study population?	Qualitative study, post-intervention survey, medical outreach mission in Repentir
		How satisfied were the participants with the intervention services?	Qualitative study, supervisory visits, facilitator videos
		How did the Malakit facilitators perceive the fit and what were the level of enthusiasm and the factors influencing their motivation?	Qualitative study, supervisory visits, facilitator videos
		What were the barriers to reach and participation?	Qualitative study, post-intervention survey, medical outreach mission in Repentir
Quality of delivery	What was the quality of message delivery?		Supervisory visits, facilitator videos
Strategies to facilitate implementation	What strategies were used to support implementation?	What were the facilitation strategies to optimize and standardize implementation adherence?	Feedback from the project implementers
Context	What internal and external contextual factors affected the implementation?	Did factors at the political, economic, organizational, geographical, or community level, and more specifically factors related to the research context, affect implementation?	Qualitative study, supervisory visits, feedback from the project implementers, facilitator videos

### Ethics

Ethical clearance has been described previously [28, 38]. They were obtained from National Ethics Committee from the countries where the project was implemented, in Brazil—Approval from the Fiocruz Ethics Committee (Opinion Number 2.831.534)—and in Suriname: Approval from the CMWO (Commissie voor Mensgebonden Wetenschappelijk Onderzoek) (Opinion Number VG 25–17)—for the Malakit study, and in Brazil—Approval from the Fiocruz Ethics Committee (Opinion Number 2.560.415)—and in Suriname—Approval from the CMWO (Opinion Number DVG-738)-, for the post-intervention survey.

### Findings of the evaluation of adherence

Adherence is defined in implementation science as the extent to which “a program service or intervention is being delivered as it was designed or written” [45].

#### Content

##### Human resources

**Fig. 3:**
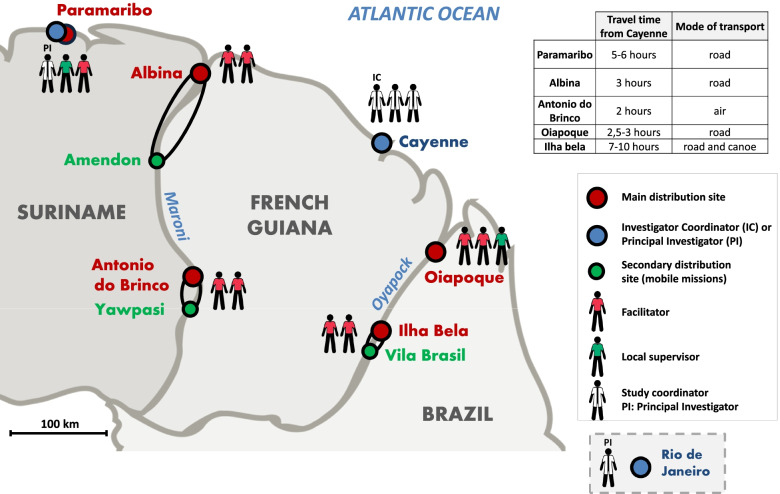
Map of the distribution sites of the Malakit intervention in Suriname and Brazil (April 2018-March 2020). Source: created by the authors

Human resources are the most important elements in most community-based approaches. The workers in this project were referred to as “facilitators” (“mediadores” in Portuguese, “médiateurs” in French). They could not be referred to as “community health workers (CHW)” as they were not active gold miners, were not chosen by the community, and were more accountable to their employer than to the community [46]. Nonetheless, belonging to or being close to the gold mining community and being fluent in Portuguese were fundamental. Having sufficient literacy skills for tablet and smartphone use was a desirable competence. Partners in both countries reported that recruitment of facilitators was difficult due to a lack of eligible candidates. Solutions to address this problem have not yet been identified. Two facilitators were assigned to each of the four border sites (see Fig. 3). In Paramaribo, tasks related to Malakit were added to the duties of the National Malaria Control Program (NMCP) staff, but after repeated failure in the quality of service, the strategy was reviewed and a full-time facilitator was assigned to this site (see Additionnal file 3). Some of the individuals who were hired were not native Portuguese speakers, despite the initial recommendations. Most facilitators had little or no knowledge of malaria and had various occupations, such as Christian pastor or boatman. The extent to which the training was implemented as planned was explained in the article on the setting-up of the project [28].

##### Details on intervention content

Additional file 3 details the adherence to content and adaptations of the key components of Malakit i.e. inclusion and training as well as kit distribution, replenishment, or re-distribution.

**Fig. 4:**
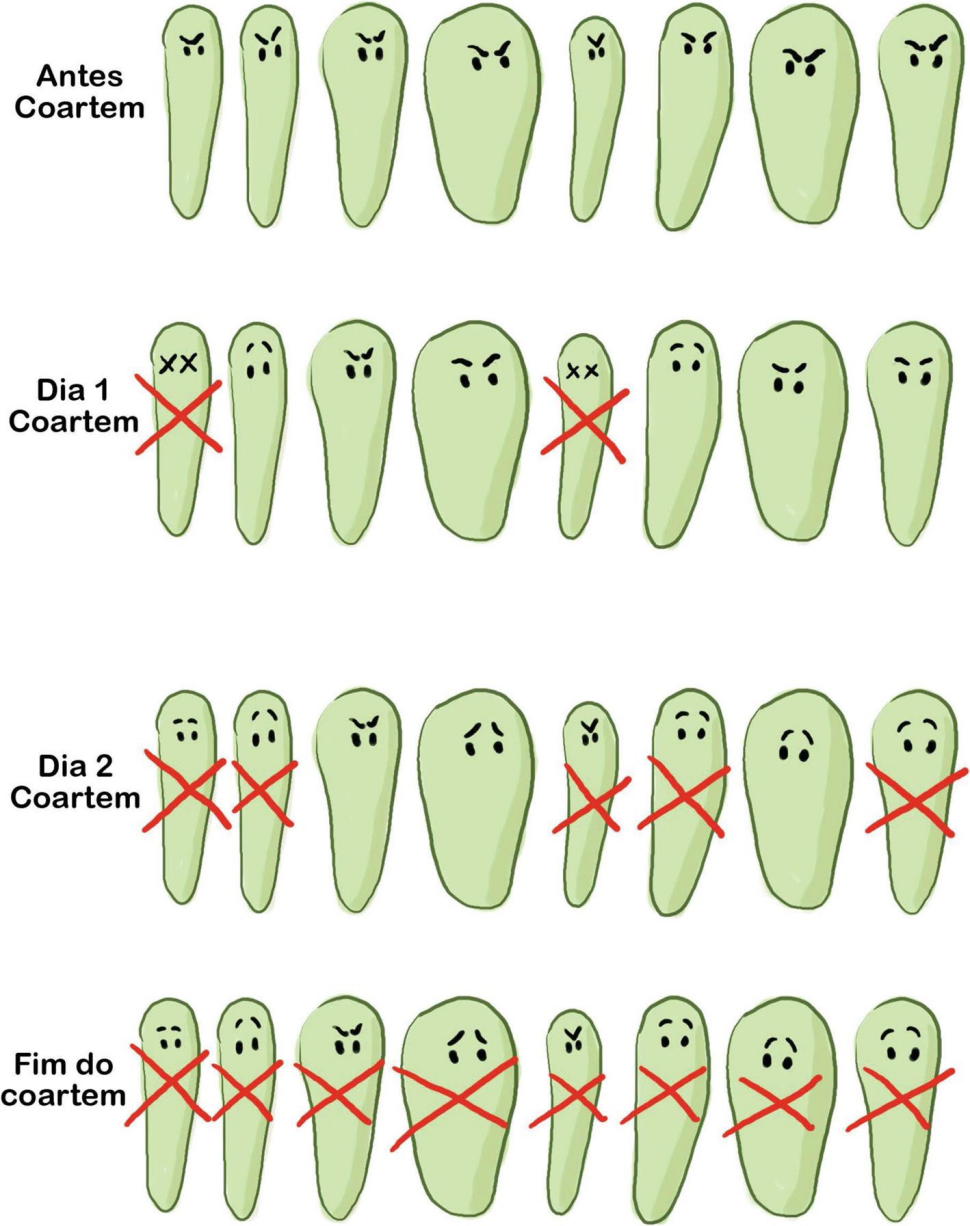
Poster illustrating the effect of the ACT on the malaria over time and the mechanism of resistance, material used during the training of participants of the Malakit intervention in Suriname and Brazil (April 2018-March 2020). Source: created by the authors

All tools created for training were systematically used, except one poster illustrating the effect of the ACT on malaria over time and the mechanism of resistance (Fig. 4), which was abandoned by some facilitators who found it redundant with the illustration of the treatment displayed on the kit [28].

#### Dose/exposure

##### Availability of the intervention

Despite a delay in implementation on the Brazilian side due to regulatory issues related to the 2018 presidential election, the project duration was not shortened thanks to the funds obtained for a six-month extension. Furthermore, the continuous presence of the facilitators at distribution sites was ensured, with the exception of the end-of-year holidays.

While the location of the distribution sites was determined at the beginning of the study, the protocol included the possibility of adapting the strategy according to the mobility of the study population. These ad hoc relocations of the intervention to additional resting sites proved to be very effective in reaching the study population but could not be repeated as often as necessary due to insufficient funding and human resources.

##### Reach/coverage

The intervention challenge of reaching the population is the same as that of assessing coverage. The findings of the qualitative study performed in 2019 revealed a good knowledge of Malakit, but a probable heterogeneity of project awareness from one site to another [42]. Data collected in a very remote and isolated mining site one year after the start of distributions (Repentir mission in June 2019) showed satisfactory penetration of the intervention. The representativeness of this sample was low (only 25 individuals with an overestimation of women (64% vs. 34% among Malakit participants)) but showed a rate of 28% (95% CI [9.1–46.7]) of the individuals encountered who had been included in the Malakit study and 60%, 95% CI [39.3–80.6]) who had heard about the project.

Finally, reaching more than 3,700 people for a population of approximately 10,000 gold miners in two years is satisfactory, given the challenging context.

### Moderators identified

Moderators are factors or mediators which can influence the degree of implementation fidelity [43].

#### Participants responsiveness

The pre-intervention survey carried out in 2015 revealed that malaria was viewed as the most important health issue in the community by gold miners. The qualitative study carried out in 2019, the year following the launch of the intervention, showed that this perception had not changed and that Malakit was considered the best solution to this health problem by the target population. The ease of use and of carriage of the kit, good contacts with facilitators, and the quality of the training were positive elements put forward. However, some participants pointed out the need to receive reminders with instructions once they were back at gold mining sites [42].

The strong acceptance and enthusiasm of participants were confirmed by the findings from the post-intervention survey and Repentir mission [38]. From the former source, 81.5% (95%CI [77.3–85.8]) of the 320 respondents acknowledged either the importance of the strategy for the population or its public health significance. Only four people expressed a negative opinion, either due to the perception that the medication supplied in the kit was not effective, the lack of usefulness due to the absence of malaria, or the need to self-administer finger pricks. The Repentir mission revealed that 12 people out of 15 who knew about the project had a very good opinion of Malakit. Only one person had a bad opinion and also thought that Coartem® was ineffective. Below are quotes from people interviewed at Repentir:

*“It is useful because we can know what disease it is and which treatment to use.”*

*“It is interesting and useful, my daughter-in-law was able to treat herself” (*gold miner who was not a participant*).*

*“It is good, good project*” (gold miner who was not a participant but had feedback from participants).

This good acceptance is also reflected by the high level of participation as half of those who knew about the project, were participating (46.1%, 95%CI [40.6–51.6] and 47%, 95%CI [18.1- 75.3] according to the post-intervention survey and Repentir mission results respectively).

The motives for not participating among individuals who had heard about the project were documented for 135 people from the post-intervention survey (Table 3). Not having the opportunity to go to a distribution site was the main reason (40/135, 29.6%). Lack of access to Malakit distribution sites, primarily due to travel costs and time, but also occasionally related to the fear of law enforcement authorities, was also the main obstacle identified in the qualitative study [42]. According to this same source, gold miners acknowledged that the time required for the training and questionnaire could be a disincentive. Facilitators also stressed the importance of making the process before handing out the kit quick, and when asked what could be improved (Additional file 2), three of them mentioned shortening the visits. One suggested reducing the training part by using more videos, and one proposed that questions be removed to shorten follow-up visits [42, 47]. The first visits lasted between 30 and 45 min, but the metadata analysis of monitoring questionnaires revealed that the median time spent on electronic data capture was five minutes, after debriefing with the participant, for both types of visits combined [34]. Reasons for refusing to be part of the study were collected by Malakit facilitators among people who were approached, in other words individuals who had the opportunity to go to a distribution site. The data are not exhaustive, come mainly from a specific site, and mainly from people who had agreed to start the training (Table 3). Lack of time was once again the main barrier that emerged.

**Table 3:** Reasons for not participating in the Malakit study (Suriname and Brazil)

Reason	People interviewed during the post-intervention survey	People approached by facilitators during the Malakit intervention (several possible answers)	
	***N* = 135** **n (%)**	**Total** ***N* = 250** **n (%)**	**People who had begun to receive training** ***N* = 140** **n (%)**
Not having had the opportunity to go to a distribution site	40 (29.6)	^a^	^a^
Unawareness of where to get a kit	7 (5.2)	^a^	^a^
Having obtained a kit by another means	6 (4.4)	^a^	^a^
Absence of facilitators at the inclusion site	3 (2.2)	^a^	^a^
Lack of time	30 (22.2)	144 (57.6)	58 (41.4)
Lack of interest in the Malakit project or lack of recognition of its utility due to perceived absence of malaria	35 (25.9)	86 (34.4)	33 (23.6)
Fear of needles	1 (0.7)	77 (30.8)	66 (47.1)
Inability to perform the RDT	^b^	7 (2.8)	7 (5.0)
Refusal to share personal information	2 (1.5)	3 (1.2)	2 (1.4)

^a^These reasons can only concern individuals who were not approached by a facilitator at a distribution site

^b^This reason can only concern individuals who were approached by facilitators. The facilitator was the person who assessed if the individual was capable of self-administering a RDT

Overall, the fear of having to self-administer a finger prick was occasionally expressed, and some facilitators reported efficient strategies to overcome this (see Additional file 3 and Table 3). All the sources of information revealed that the inability to perform a self-test – excluding the fear of needles – and the reluctance to share personal data, which were anticipated as potential barriers, were rarely reported. Facilitators confirmed that distrust was generally overcome and tended to decrease over the course of the project [42].

#### Facilitators responsiveness

The qualitative study mentioned the perceived importance and relevance of the project from facilitators, and even a pride in doing their work, especially among those who had been gold miners before [42]. Three facilitators reported during supervisory visits a wary attitude among the gold miners, attributed to their accent betraying the fact that they were not Brazilian, but which rapidly dissipated after the project objectives were presented.

The feeling of being useful and part of an innovative project which they believed in was also an important incentive pointed out by the vast majority in their self-shot videos [47]. Three facilitators also reported that what they liked most about their work was acquiring new knowledge. Continuous capacity building is probably crucial to maintaining human resources and sustaining their motivation over time.

#### Quality of message delivery

Sometimes, less can be more. The shortcoming encountered, mainly at the beginning of the project, was an excess of information or inaccurate information, rather than a lack of information given to participants by facilitators. Adding too much detail may dilute the important information or make it confusing and eventually become detrimental to training. For example, one facilitator described the drug primaquine included in the kit as abortifacient when explaining that it should not be taken by pregnant women, a description that could lead to misuse of the drug for that very purpose. While speaking about risks, instead of only explaining the danger of Coartem® in patients with heart problems, a facilitator also mentioned the risk for Artecom®, the main antimalarial drug found on the black market. This may have undermined the message about avoiding under-the-counter medications. As time went by, the talk was well mastered. The facilitators confirmed that they adjusted the time spent explaining, the stress on specific messages, the number of repetitions, and the vocabulary according to the audience and its availability, as the training was highly interactive.

Overall, facilitators without a health worker background revealed a better ability to tailor the message to the needs of the study population, according to observations in the field.

#### Facilitation strategies

One principal investigator located in Rio de Janeiro and one coordinator in Oiapoque were responsible for the Brazilian sites. A single person was both coordinator and principal investigator in Suriname. However, these assignments were carried out in addition to their usual work. Due to the distance between the project implementation team in Cayenne and the distribution sites, it was decided that in each country, a supervisor ranking above the facilitators would be hired, to supplement the regular visits of the sponsor team and continuous monitoring of the data collected by the facilitators (see Fig. 3). In the long run, direct interaction between the sponsor team and facilitators in the field on both border rivers proved to be more convenient. Indeed, except for two sites, distance was also an issue for frequent on-site supervisory visits. Non-availability of the supervisors due to lack of time on one border, and difficulties in hiring a permanent supervisor on the other, contributed to poor ongoing training. Nonetheless, the ambiguity of supervisory role of the sponsor team in Cayenne without hierarchical relations was sometimes confusing for facilitators.

To counteract distance issues, facilitators were provided with field reports. The purpose was to improve and homogenize practices by capitalizing on experiences in the field, reiterating important points, and formalizing certain guidelines. This information was provided digitally via an instant messaging group and on paper. Despite efforts to summarize instructions and make them more palatable with the addition of diagrams and pictures, facilitators showed variable interest in these reports. One-to-one direct debriefings, either in person or via instant messaging, had a greater impact. Oral culture seemed to prevail over written culture among facilitators.

#### Context

##### Factors influencing reach and participation

Several contextual factors can influence mobility and thus the frequentation of resting sites. The following are the main ones identified: 1) French police operations at mining sites in French Guiana and the presence of the Brazilian army on the Brazilian border; 2) Seasons and periods of the year (for example, greater mobility during end-of-year holidays); 3) Gold mining activity depending on the location of gold veins and rushes following rumors of new discoveries (in Portuguese, *fofoca*), and indirectly, the presence of armed gangs (*facções*); 4) Occupation at gold mining site, mobile activity (e.g. traveling vendors, transport providers, and porters) versus non-mobile activity (e.g. gold miners, shop owners, machine owners)*.*

The ability and willingness of potential participants to spend their time on Malakit training was more or less significant depending on the location of the distribution site (see Table 4). At distribution sites where gold miners were just passing through before reaching their final destination (e.g. Albina) and/or where departure by boat to the gold mining sites could be sudden and thus where gold miners were on the lookout (e.g*.* Vila Brasil), the time required for inclusion was a barrier, since obtaining a malakit was not a priority. On the other hand, participants who lived at the resting site (Antonio do Brinco, Ilha Bela, Oiapoque) and had no “competing activity” showed a much greater engagement in the intervention. Facilitators also reported better availability of participants at temporary distribution points, during one-off missions.

**Table 4:** Characteristics of distribution sites of the Malakit intervention in Suriname and Brazil (April 2018-March 2020)

Name of distribution site	Number of full-time facilitators	Facilitator turnover	Type of premises	Location	Distance from referral facility	Characteristics
**Suriname**						
Albina	1.5	No	Small prefabricated structure Already a malaria clinic before the project start	Logistics base, near shops and gold miner resting site	On site, a facilitator is also a malaria test and treat worker (MSD)	Most gold miners are there in transit for a very short period of time First mining sites are very close
Antonio do Brinco	2	No	No fixed facility	Mobile facilitators in a gold mining village, or in a church (when the water level prevents walking)	Malaria clinic with a MSD within the village, but not used by the facilitators	Most gold miners live and stay there for quite long periods of time First mining sites are very close
Paramaribo	1 (initially 6 part-time)	Yes	Office in a hotel	Brazilian neighborhood in the Surinamese capital, hotel frequented by gold miners. The facilitator also works in the hotel	TropClinic is 5 min away by taxi	Short stays for the majority of gold miners. Gold miners working specifically in French Guiana pass through less frequently
**Brazil**						
Oiapoque	2	Yes	Fixed office in an apartment	Small town, within the goldminers’ neighborhood	Health centers and a hospital in the town, but not in the neighborhood	Departure point for gold mining sites, but no sites in the vicinity. Many gold miners have a permanent home in the town
Ilha Bela	2	Yes	Shack	Within a small spontaneous settlement of gold miners	No malaria care on site. Oiapoque, located 4–5 h away by boat, is the closest place miners are referred to	Isolated villages First mining sites are very close Facilitators are based in Ilha Bela and go to Vila Brasil twice a week
Vila Brasil			Health center	Village located 30 min from Ilha Bela		

During the project period, the incidence of malaria decreased at the gold mining sites and in the region (decrease partly attributable to the project [38]. This decrease could lead to the perception that malaria no longer exists followed by a diminished interest in obtaining a malakit. Towards the end of the implementation phase, facilitators at one particular site reported several cases of people who felt that the kit was not relevant for them, as they considered that malaria was no longer present at their mining site.

##### Potential economical moderator

The relatively high market value of the kit itself (more than two grams of gold according to gold miner testimonies i.e. about 85 USD) represented several risks such as resale [48]. Despite close verification of stock flows, the intermittent presence of supervisors in the field made it impossible to ensure that no kits were resold by facilitators.

##### Influence of the research context

The context of a research is different from a public health intervention as measurement can disturb the object measured. Although the degree of pragmatism in this intervention was high on the pragmatic-explanatory continuum, it was not implemented in fully real-world settings [9]. For example, the instruction given to facilitators not to judge participants for misusing kits (e.g. by sharing them), in order to encourage them to tell the truth about practices, may have led participants to feel that sharing the kit was acceptable. Moreover, the continuous and longitudinal collection of data carried out by the facilitators as part of a research project was more extensive than it would have been if a public health intervention were being monitored. Despite efforts to limit the number of questions and to avoid sensitive topics (e.g. questions were asked on past whereabouts only, not on future destinations), the questionnaires may have been a source of suspicion for a community constantly on their guard due to their illegal and clandestine status. Conversely, the multitude of partners from different countries and the logos displayed on easels and facilitators’ vests were a source of trust for the participants [28].

The difference of diagnostic method between Malakit; i.e. CareStart™ Malaria (Pan) and those provided at malaria clinic in Suriname, i.e. Sd Bioline Malaria Antigen P.f/P.f/P.v® and microscopy, and Brazil, i.e. microscopy, sometimes led to divergent results. Because of the large number of persons tested, mostly asymptomatic, the frequency of such discordant diagnoses seemed high. An investigation using PCR as a gold standard found a false-positive proportion of 1.72%, consistent with an expected false positive rate of 2.4% for a reported specificity of 97.6% and in a low prevalence setting, and a Positive Predictive Value of 40%, consistent with the low PCR prevalence of 5.3% measured during the post-intervention survey [38]. Routine diagnosis was performed on Malakit participants only in case of symptoms or positive Malakit RDT on the Surinamese side and systematically on the Brazilian side. Despite a common procedure to address this problem agreed on among the stakeholders concerned, confusion among participants and decreased confidence in the Malakit RDT or in routine care were reported. Moreover, due to its success, the intervention may have competed with malaria routine care despite the complementarity of the two. A feeling of competition was expressed by some health care workers, which was then dissipated thanks to improved communication.

### Discussion on adherence, quality of delivery and its moderators

Based on the observation data, it is possible to assert that the content of the messages and the way they were transmitted complied satisfactorily with what was planned, even if rectifications were necessary at the beginning.

Dane and Schneider suggest [49] that lack of confidence or experience, as well as not being professional – i.e. being a paraprofessional or lay person –, are predictors of poor program integrity in preventive intervention research. Conversely, in a study assessing the fidelity of implementation of malaria care for children by community health workers (CHWs) in Nigeria, adherence to the diagnostic, treatment, and counseling protocol by CHWs was found to be equal or higher to that of the medical staff who served as gold-standard comparators, and was not related to age, level of education, or primary occupation. In the Malakit intervention, previous experience in health care or health mediation did not seem to be an asset – also since, compared to other interventions, no clinical evaluation was performed –, and overconfidence was actually a barrier to compliance with what was planned, to the point where facilitators had to be replaced (see Additional file 3, “Terms and conditions to be included”). This is consistent with WHO’s finding that CHWs can be men or women, young or old, literate or illiterate, as long as they blend into the culture of the community and ensure its acceptance [46].

Ongoing training is a recommended practice, but can be linked to dissatisfaction when format, frequency, quality, etc. are judged inappropriate or insufficient by CHW [50]. Facilitators did not express such discontent, despite many opportunities to do so. The use of mobile technologies in particular was quite well accepted [34]. In low and middle income countries, they are increasingly seen as an opportunity to better train and improve worker performance remotely [51, 52]. This approach, also known as “Mobile Learning for Development”, is the subject of recent studies that concluded that there is a need for further research to better assess and adapt approaches [53, 54]. In Kenya, in a very similar manner to the Malakit intervention, an intervention included a WhatsApp group to strengthen “supervision, professional development and team building”, and also found that quality assurance, information sharing, and the creation of a supportive environment were useful [55]. More broadly, social interaction and peer assessment have been found to be associated with better guideline implementation and clinical practice change [56, 57]. In the present project, the peer-to-peer form of supervision within the WhatsApp group was not observed. Facilitators in the two countries knew each other slightly or not at all, due to the limited number of joint training sessions or meetings (all of them needed a visa to enter French Guiana). That is why they may not have felt comfortable enough to ask questions and share difficulties, and tended instead to share successes. In-person peer supervision, which at one point was considered, can be a way to further foster performance, but could not be implemented.

The geographical distance issues identified here as a main constraint to implementation and monitoring may be encountered in other contexts involving several countries and should be addressed. In addition to instant messaging debriefings, field supervision and refresher training, which are very time-consuming when two days are needed to reach a site, should be assigned to someone dedicated solely to those tasks. This person should actively collaborate in designing and developing training contents and data collection tools with the principal investigators. The development of refresher training tools for facilitators using a participatory approach – as used for participant training tools –, in order to adapt content to their literacy and needs, could also alleviate distance issues.

Constant and long-term efforts to maintain quality are essential to adapt to evolving contexts, including beyond scale-up. Indeed, while resources allocated for research can be sufficient to ensure integrity, for example through continuous in-person and remote supervision, decrease in fidelity is more likely when interventions are adopted and sustained [49]. Further qualitative research is planned during the sustainability phase in Suriname to add and or improve communication tools and ways of delivering messages to enhance quality of delivery.

### Discussion on reach/coverage and its moderators

Although coverage was acceptable after two years of intervention, it could have been improved by better allocating funds and resources to adjust to the gold miners’ mobility in a timely manner, especially given the increasing heterogeneity of malaria transmission among gold mining regions. While the penetration of the intervention was very good in a remote gold mining region where traveling to reach a distribution site is costly and time consuming, lack of access remains a barrier to better coverage, as mentioned in the qualitative study and the quantitative results of the post-intervention survey [38, 42].

The excellent appropriateness – defined as “perceived fit of the innovation to address a particular problem” already observed during the feasibility study as well as during participatory development of communication tools, good “adoption” or “uptake” by the study population and finally great acceptability definitely boosted reach [58]. Adjusting the length of training could be a mediator to increase acceptability, especially at certain distribution sites where time is a limiting factor. Furthermore, the findings underline the importance of factors that contribute to the population’s trust in the project, especially with wary communities. Research requirements in particular can negatively impact the community’s perception of the project, which should not be underestimated. Although the strategy was still at the experimental stage, articulation with existing care services should have been further developed to avoid competition being felt instead of complementarity. Finally, diminution of malaria prevalence may imply decrease of participant responsiveness more or less depending on the place of distribution [58]. Maintaining community uptake could be achieved by expanding services offered by facilitators, as seen in Myanmar, where the management of non-malaria febrile illnesses and the referral of severely ill patients complemented “malaria only” CHW prerogatives [59].

### Strengths, biases and limitations

While the main defect of the present assessment is the absence of quantitative indicators for content adherence, the main strength is the regularity of supervisory visits throughout the project and not only during specific periods. The distinction between the “core components” and adaptable elements of an intervention can only be discerned through practice and mispractice over time as the intervention is more widely deployed and replicated in other contexts, as explained in the Consolidated Framework of Implementation Research [60]. Since Malakit was an innovative strategy, several choices and adaptations were made during the implementation itself. Thus, the objective of this article was not to create fidelity measures to assess an evidence-based intervention, but to capitalize on this unprecedented field experiment to contribute to future process evaluation or implementation research on the strategy. This is why no proper observation grids were designed to assess adherence to content or quality of delivery and why program differentiation, which is apart from fidelity, was not performed [43].

A workshop bringing together all facilitators and supervisors and led by an external assessor was planned in April 2020 but was cancelled due to the COVID-19 pandemic. The self-recorded videos requested of facilitators to replace their presence at the final meeting of the project were another way to give them a voice. Although this format was not anonymous, which may have inhibited the free expression of opinions, the videos made it possible to confirm or complete information on moderating factors, such as the influence of the length of the training on participation.

One limitation of the post-intervention survey was an over-representation of people frequently traveling to resting sites linked to an overestimation of the coverage. Lack of knowledge on the study population size and flows also made it difficult to assess the estimation of coverage based on distribution figures. The findings of the medical outreach mission in a hard-to-reach gold mining site provided some information on penetration despite the small size of the sample. In both of these quantitative data collections, biases were also over-representation of health-conscious individuals and expected response bias with over-reporting of positive opinion on the project. The qualitative study carried out by an external assessor allowed for increased freedom of expression and to some extent made it possible to alleviate this last bias [38].

Although it is not independent and external, feedback from players who were engaged in the protocol (intervention design and evaluation) and tools development, training, and close field supervision, can constitute in-depth information to complete qualitative and quantitative data collection.

## Conclusions

These findings supplement the previously published effectiveness results by reporting on what was actually implemented and the moderating factors of the implementation, thereby strengthening the overall evaluation of the intervention [38]. Satisfactory compliance with what was planned, good responsiveness of the participants and improvements to be done to reach the population are the main points observed. In addition, comparison of the protocol with reality on the ground highlights considerations that will be essential for the sustainability, applicability and transferability of the strategy in other contexts [35, 36, 61].

## Supplementary Information

**Additional file 1.**

**Additional file 2.**

**Additional file 3.**

## Data Availability

The datasets used and/or analysed during the current study are available from the corresponding author on reasonable request.

## Abbreviations

ACTs: Artemisinin-based combination therapy
CHW: Community health workers
CMWO: Commissie voor Mensgebonden Wetenschappelijk Onderzoek
NGO: Non-government organization
NMCP: National Malaria Control Program
RDT: Rapid diagnostic tests
SWOS: Foundation for the Advancement of Scientific Research in Suriname
