# Supplementary material for: Implementation of a novel malaria management strategy based on self-testing and self-treatment in remote areas in the Amazon (Malakit): confronting a-priori assumptions with reality
Source: BMC Public Health. 2022 Apr 15;22:770. doi: 10.1186/s12889-022-12801-0 (PMC9012048; doi:10.1186/s12889-022-12801-0)
Supplement: Supplementary file 1 — Additional file 1. [file 12889_2022_12801_MOESM1_ESM.docx]

Both Suriname and French Guiana have experienced a drastic increase in malaria incidence since the early 2000s followed by a sharp drop in the number of malaria cases starting in 2005 (1,2). The peak was reached in 2005 in French Guiana with 4,479 cases of malaria reported; only 256 were reported in 2016 (3,4). In Suriname, the number of cases went from 10,713 in 2003 to 335 in 2016 (5,6).

This overall decrease was mainly due to the generalized use of ACT (artemisinin-based combination therapy) and successful implementation of prevention and control interventions in Suriname (6,7). However, while the number of locally-acquired cases fell in Suriname and on the Maroni river in French Guiana until 2015, a rise was observed in the inland areas and the eastern region of French Guiana (border with Brazil) and in the number of imported cases in Suriname (1,2,8).

Several clues indicated that this situation could be attributed to illegal gold mining in French Guiana and the mobility of the population engaged in that activity, which was not targeted by any control measures (1,4,6,9). The Orpal study (also referred to as the pre-intervention survey in the main text), carried out in 2015 among 421 gold miners included at staging areas along the Maroni river, confirmed this hypothesis. It revealed a prevalence of 22.3% of plasmodium carriage; 84% of the carriers were asymptomatic. Rates were heterogeneous, ranging from 3.8% to 46.4% depending on the location, with the region most affected located between Maripasoula (village on the Maroni river) and Saül (inland village) (10).

In Brazil, malaria is endemic almost exclusively in the Amazon region. The country has experienced a total decrease in cases of over 75% in 35 years, allowing it to launch an eradication plan in 2016. However, this reduction was more modest in Amapá, especially in the municipality of Oiapoque on the border with French Guiana, which recorded a reduction of 55.1%.  The number of cases there decreased from 3,877 in 2003 to 1,236 in 2015, with a peak of 6,593 cases in 2007. Once again, the flows of illegal gold miners over the border constitute a key factor for malaria in the municipality. However, the proportion of imported cases has clearly fallen, going from 67.7% between2003 and 2007 to 32.69% between 2008 and 2015 (p< 0.001) (11–13).

1. Musset L, Pelleau S, Girod R, Ardillon V, Carvalho L, Dusfour I, et al. Malaria on the Guiana Shield: a review of the situation in French Guiana. Memorias do Instituto Oswaldo Cruz. 2014 Aug;109(5):525–33.

2. Hiwat H, Martínez-López B, Cairo H, Hardjopawiro L, Boerleider A, Duarte EC, et al. Malaria epidemiology in Suriname from 2000 to 2016: trends, opportunities and challenges for elimination. Malar J [Internet]. 2018 Nov 12 [cited 2019 Mar 13];17. Available from: https://www.ncbi.nlm.nih.gov/pmc/articles/PMC6233553/

3. Ardillon V, Eltges F, Chocho A, Chantilly S, Carvalho L, Flamand C, et al. Evolution de la situation épidémiologique du paludisme en Guyane de 2005 à 2011. Bulletin de Veille Sanitaire - Cire Antilles-Guyane. 2012 Feb;1–2:5–11.

4. Andrieu A, Ardillon V, Carvalho L, Petit-Sinturel M, Quet F, Bourdillon F. Surveillance du paludisme. Bulletin périodique : novembre 2017 à janvier 2018. Le point épidémiologique CIRE Guyane. 2017 Jan;N°1/2018:3.

5. Breeveld FJ, Vreden SG, Grobusch MP. History of malaria research and its contribution to the malaria control success in Suriname: a review. Malaria journal. 2012;11:95.

6. van Eer ED, Bretas G, Hiwat H. Decreased endemic malaria in Suriname: moving towards elimination. Malar J. 2018 Jan 30;17(1):56.

7. Breeveld FJ, Vreden SG, Grobusch MP. History of malaria research and its contribution to the malaria control success in Suriname: a review. Malaria journal. 2012;11:95.

8. Labadie-Bracho MY, van Genderen FT, Adhin MR. Malaria serology data from the Guiana shield: first insight in IgG antibody responses to Plasmodium falciparum, Plasmodium vivax and Plasmodium malariae antigens in Suriname. Malaria Journal. 2020 Oct 8;19(1):360.

9. Andrieu A, Ardillon V, Carvalho L, Petit-Sinturel M, Quet F, Bourdillon F. Surveillance du paludisme. Bulletin périodique : octobre à décembre 2016. Le point épidémiologique CIRE Guyane. 2017 Jan;N°1/2017:3.

10. Douine M, Musset L, Corlin F, Pelleau S, Pasquier J, Mutricy L, et al. Prevalence of Plasmodium spp. in illegal gold miners in French Guiana in 2015: a hidden but critical malaria reservoir. Malar J. 2016;15:315.

11. Suárez-Mutis MC, Martinez-Espinosa FE. Malária. In: Coura JR & Pereira NG. Fundamentos das Doenças Infecciosas e Parasitárias. Elsevier. Rio de Janeiro; 2019. 279–293 p.

12. da Cruz Franco V, Peiter PC, Carvajal-Cortés JJ, dos Santos Pereira R, Mendonça Gomes M do S, Suárez-Mutis MC. Complex malaria epidemiology in an international border area between Brazil and French Guiana: challenges for elimination. Trop Med Health. 2019 Apr 11;47(1):24.

13. Gomes M do SM, Menezes RA de O, Vieira JLF, Mendes AM, Silva G de V, Peiter PC, et al. Malaria in the borders between Brazil and French Guiana: social and environmental health determinants and their influence on the permanence of the disease. Saude soc. 2020 Jun 5;29:e181046.
