# Supplementary material for: Implementation of a novel malaria management strategy based on self-testing and self-treatment in remote areas in the Amazon (Malakit): confronting a-priori assumptions with reality
Source: BMC Public Health. 2022 Apr 15;22:770. doi: 10.1186/s12889-022-12801-0 (PMC9012048; doi:10.1186/s12889-022-12801-0)
Supplement: Supplementary file 2 — Additional file 2. [file 12889_2022_12801_MOESM2_ESM.docx]

# Questions for facilitators

1. Can you introduce yourself?

2. Can you describe your tasks/activities within the Malakit project?

3. What did you enjoy most about working as a Malakit facilitator?

4. What did you least like about working as a Malakit facilitator?

5. In your opinion, what was the easiest and most difficult part of the training to make the participants understand or master?

a. What adaptations/tips/customizations of the training did you make to improve it?

6. *For those who have worked in different sites/ mobile offsite missions*: What differences did you notice between the sites?

a. Did these differences impact the Malakit intervention?

7. Have you noticed a change in the reputation of the project over time, in the attitude of the population towards the project?

8. What are the strengths and weaknesses of the strategy as it was implemented? Enabling factors? Barriers?

9. What recommendations would you give to improve it?

10. Free Comments
