# Supplementary material for: Implementation of a novel malaria management strategy based on self-testing and self-treatment in remote areas in the Amazon (Malakit): confronting a-priori assumptions with reality
Source: BMC Public Health. 2022 Apr 15;22:770. doi: 10.1186/s12889-022-12801-0 (PMC9012048; doi:10.1186/s12889-022-12801-0)
Supplement: Supplementary file 3 — Additional file 3. [file 12889_2022_12801_MOESM3_ESM.docx]

| **WHAT WAS PLANNED AND ASSUMPTIONS** | | **WHAT WAS IMPLEMENTED AND ADAPTATIONS** |
| --- | --- | --- |
| **Participant recruitment** | | |
| The recruitment strategy was to offer a malakit along with training to any eligible person passing by.  Instructions were to inform each potential participant that the process would take one hour and involved signing a consent form and self-administering a finger prick for self-test training. | Inclusion was usually performed right after eligibility screening. An appointment was only given when it was not possible to include the person right away.  Some facilitators were more comfortable announcing a process length of 30 or 45 minutes rather than an hour, which was perceived to be too long. | |
| **Number of participants per session** | | |
| Facilitators were instructed to perform the **inclusion process in pairs** for **one to four participants at a time.** | With time and practice, the facilitators were able to conduct the inclusion process on their own for up to four participants. The number of participants attending theoretical training sessions was higher in special circumstances, such as off-site outreach missions. However, experience showed that facilitators cannot monitor self-testing for more than two people at a time. That practice was well followed, as observed during supervisory visits and as reported by the facilitators. Shifting to single-participant training may allow further tailoring to individual needs and consequently improve learning efficiency. | |
| **Terms and conditions to be included in the study** | | |
| The decision to provide **a single kit**  to each participant and instruct them  **not to give it away or share it** was driven by the desire to avoid the illegal practice of medicine and resale on the black market. | | Data and stock monitoring as well as supervisory visits confirmed that **facilitators handed out one kit per person per visit.**  Screening of potential dishonest behavior among participants was included in the analysis plan. |
| Ability to use the kit was an inclusion criterion. It was decided that facilitators would assess this ability by **observing the practical performance of a self-test** with the help of a checklist of the  critical steps to follow. | | **Supervised self-testing was performed systematically**. Facilitators provided guidance when needed, but oral help only was given. If a person was apprehensive about pricking themselves, the facilitator was allowed at most to gently guide the participant's hand holding the needle closer to the participant's finger.  Despite prior warning on the importance of teaching self-testing to participants, two facilitators were observed handing out a kit without asking participants to perform a RDT on themselves, without understanding why this was an issue. The sponsor team agreed with the on-site coordinator to replace these facilitators, who then no longer worked for the project. |
| Understanding the proper use of medications was an inclusion criterion. **Facilitators would verify that participants understood using a standardized teach-back method** at the end of the training session, i.e. the participant had to be able to summarize all key points on their own: *taking antimalarial drugs only after a positive diagnosis, knowing the medication dosage.* | | During the first supervisory visits, facilitators only relied on their perception to determine the participants’ understanding and considered the teach-back method to be pointless. **After several debriefings, the procedure’s objective was understood and became systematic.** |

| **Messages delivered** | |
| --- | --- |
| Explanation on ACT dosage: the instructions on the kit indicated a three-day regimen, and when facilitators would check participants’ understanding of the Coartem® course, the expected answer was three days. | As the kit indicates a first dose in the morning**, people may have been misled into starting treatment the next morning, rather than starting treatment immediately.** On the other hand, even though the facilitators stressed the importance of taking the entire treatment, **participants may have been confused by the fact that the medication is taken over four days when it is started in the evening.** Facilitators were made aware of this issue and adapted their explanation accordingly. |
| Instructions on the use of the tests were: only re-test if the result is invalid, and under no circumstances in order to verify if the treatment has worked. | As many participants reported using several RDTs within a short period of time, it was decided to extend the following message to all distribution sites: “*If your test is negative,* ***if symptoms*** ***persist*** *and no malaria medication has been taken, do not take Coartem*® *and* ***repeat the test after one day****”*, as advised in studies on self-administered RDT (1,2)^[[1]](#endnote-1)^. |
| *“Start the treatment and go to a health center as soon as possible”* was the message to be given in case of signs of severe illness, vomiting, or if the patient was on heart medication, which could be linked to a long QT, a contraindication to using Coartem®. | At one distribution site where it was possible to consult a health care professional before going into the forest, additional advice was given: “*If you take heart medication, before going to the gold mines, check with a doctor to see if you can take Coartem*®”. |
| **Terms and conditions of follow-up visits** | |
| Collecting data from a sufficient number of follow-up visits was important, as the continuous evaluation of the intervention was based on it.Therefore, it was important to avoid barriers that could prevent participants from attending a follow-up visit**. Thus, it was decided that replenishment or delivery of a new kit would not be contingent on the restitution of used kits**. Indeed, kits can be lost, stolen, or destroyed during police operations to fight illegal gold mining activity. However, refusal if the person obviously came back too frequently was left to the facilitators’ judgment. | **The facilitators handed out kits to people who returned several times, even to people who had not used their kits** because they had been lost, given away, stolen, or destroyed during French police operations to combat illegal gold mining (Harpie operations). They did not refuse to donate a kit on suspicion.  **However, monitoring of the data detected occasional suspicious behavior**. |
| The facilitators were tasked with assessing correct use of the kits and refreshing participants’ knowledge if necessary when they came back, before delivering a new kit. **Questions about kit use were thus included in the data collected from the outset of the project.** | **A first adjustment was made to the questionnaire shortly after the field launch**: **additional questions were added to assess what participants remembered from previous training**. The aim was twofold: 1) to better assess the level of knowledge of each individual participant and identify the need for refresher training, 2) to obtain a new evaluation indicator, particularly useful when participants did not use the kit.  **A second adjustment was made** to the follow-up visit process: **systematic self-testing by the returning participant** was added to ensure that the person could not pretend to have already been included and trained. It also offered a new opportunity to practice self-testing. |

1. 1. Maltha J, Gillet P, Jacobs J. Malaria rapid diagnostic tests in travel medicine. Clin Microbiol Infect. 2013 May;19(5):408–15.

   2. Berthod D, Rochat J, Voumard R, Rochat L, Genton B, D’Acremont V. Self-diagnosis of malaria by travellers: a cohort study on the use of malaria rapid diagnostic tests provided by a Swiss travel clinic. Malar J. 2017 28;16(1):436. [↑](#endnote-ref-1)
